# Modeling Carbon Balance and Sugar Content of *Vitis vinifera* under Two Different Trellis Systems

**DOI:** 10.3390/plants10081675

**Published:** 2021-08-15

**Authors:** Linda Salvi, Eleonora Cataldo, Sofia Sbraci, Francesca Paoli, Maddalena Fucile, Eleonora Nistor, Giovan Battista Mattii

**Affiliations:** 1 Department of Agriculture, Food, Environment and Forestry (DAGRI), University of Florence, 50144 Florence, Italy; linda.salvi@unifi.it (L.S.); eleonora.cataldo@unifi.it (E.C.); sofia.sbraci@unifi.it (S.S.); francesca.paoli@unifi.it (F.P.); maddalena.fucile@unifi.it (M.F.); 2 Department of Horticulture, Banat University of Agricultural Sciences and Veterinary Medicine, 300645 Timișoara, Romania; nisnoranisnora@gmail.com

**Keywords:** grapevine management, climate change, photosynthesis, yield, quality, STELLA software, vertical shoot positioning trellis, single high wire trellis

## Abstract

Environmental factors might influence the carbon balance and sugar content in grapevine. In this two-year research, the STELLA software was employed to predict dry matter accumulation in Sangiovese vines, comparing the traditional vertical shoot positioning (VSP) and the single high wire (SHW) trellis systems. Every week, vegetative, eco-physiological and grape quality parameters were collected for 15 tagged vines per trellis system to set up the software. Significant differences in photosynthesis were recorded in 2014, with higher values in VSP (23–25% more). Shoot growth was significantly higher in VSP (20–25% more), whereas higher dry matter (30%) and yield (9–11% more) were detected for SHW. At harvest, berry composition suggested a slower ripening in SHW compared to VSP, which was linked to the shading of clusters in SHW. Finally, for the first time, linear regressions were found between measured berry sugar content and STELLA-estimated dry matter (R^2^ = 0.96 in VSP; R^2^ = 0.95 in SHW). This latter evidence allowed the estimation of berry sugar content, showing this software to be a practical tool to support winegrowers in decision making. Other studies are already underway to calibrate and validate the model for other varieties, training systems and environments.

## 1. Introduction

In the next decades, the climate will be characterized by increasing temperatures and concentrations of greenhouse gases in the atmosphere, as well as by changes in precipitation patterns [1].

Grapevine, one of the most widespread perennial crops in Europe [2], provides an example of an agricultural system that is highly sensitive and adaptive to changes in climate conditions [3,4].

High temperatures, in combination with water deficit, influence vine physiology and accelerate berry ripening processes, resulting in unbalanced wines with a high alcoholic content and low polyphenolic complexity [5,6,7,8]. Moreover, the rise of the atmospheric CO_2_ concentration, through its influence as the source of carbon for photosynthesis, will have a significant stimulatory effect on grapevine vigor and yield, affecting sugar content and secondary metabolites [9,10,11].

Hence, the evaluation of the carbon balance in grapevine is of great importance, especially in premium-wine producing areas, where the proper management of the vineyard represents a sustainable method to reach optimal grape quality [6,12,13,14].

The quantification of carbon dioxide depletion through photosynthesis can be achieved at the “whole-plant level” by enclosing whole plants in large air flux chambers coupled to a gas exchange analyzer [15,16,17,18] or at the “single organ level” using conventional gas exchange systems [19,20,21,22,23,24].

However, a realistic and less time-consuming approach, in the long term, to the estimation of the plant supply–demand balance for carbohydrates may be offered by modeling, which is widely used to measure climate impacts on annual crops [25,26] but is less often applied to perennial crops as *Vitis vinifera* [6,27,28,29].

Among the existing models, the STELLA software was proposed at the beginning of 1990 to forecast crop growth and development in apple trees [30,31]. More recently, this user-friendly software was validated for grapevine in order to predict the daily carbon balance and dry matter accumulation of canopies [27,28,32,33], but it has never been exploited to predict the grape quality parameters that are strictly related to the carbon cycle, such as berry sugar content. For these reasons, the objectives of this research were (1) to estimate the dry matter accumulation and validate the STELLA software in cv. Sangiovese grapevines trained with vertical shoot positioning and with a single high wire and (2) to implement the software in order to predict the sugar content accumulation in the berries.

With this goal, vegetative, eco-physiological and grape quality parameters were measured for vines with destructive methods to run STELLA and verify the reliability of the software outputs.

Since dry matter accumulation can be strongly influenced by the season [34] and by the training system of the vines [35], this experiment was conducted over two growing seasons, comparing the widespread traditional vertical shoot positioning trellis with the single high wire trellis—an ancient free canopy system that has recently started to be taken into consideration and prevents berry overheating thanks to its falling vegetation [36,37]. Therefore, the present study had the further purpose of helping to clarify if the single high wire trellis can be considered as a technological solution for a viticulture that is severely challenged by global warming.

## 2. Results and Discussion

### 2.1. Vineyard Microclimate, Leaf Gas Exchange and Canopy Light Interception

**Figure 1:**
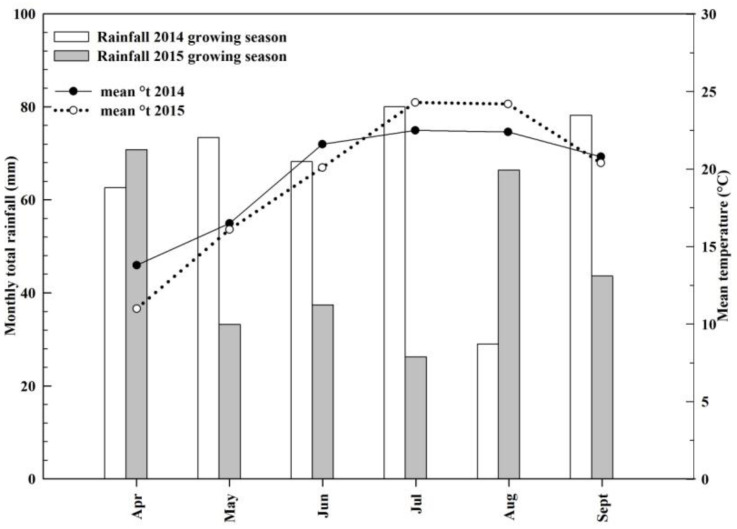
Monthly total rainfall (mm) and mean temperature (°C) over the 2014 and 2015 growing seasons (from April to September).

The climate in our study period was typical of the Mediterranean region, although some differences in rainfall pattern were detected in the two years of research (Figure 1): 2014 (391.4 mm rain/growing season) was characterized by an even distribution of rainfall, especially during summer, whereas a dry period in May, June and July was observed in 2015 (277.6 mm rain/growing season).

Growing degree days (GDDs) were 1938 °C in 2014 and 2119 °C in 2015. The higher value in 2015 was the result of higher temperatures in July and August (Figure 1).

As they were affected by the differences in meteorological conditions between the two seasons, sprouting occurred on 1 April 2014 and 28 March 2015, and flowering (50% cap fall) occurred on 3 June 2014 and 27 May 2015. The onset of veraison (50% of colored berries) was recorded on 26 July 2014 and 22 July 2015; grapes reached maturity and were harvested on 11 September 2014 and 22 September 2015. The harvest in 2014 was early than that in 2015 due to the meteorological conditions, especially rainfall in September, to prevent the onset of rot attack (*Botrytis cinerea*).

**Figure 2:**
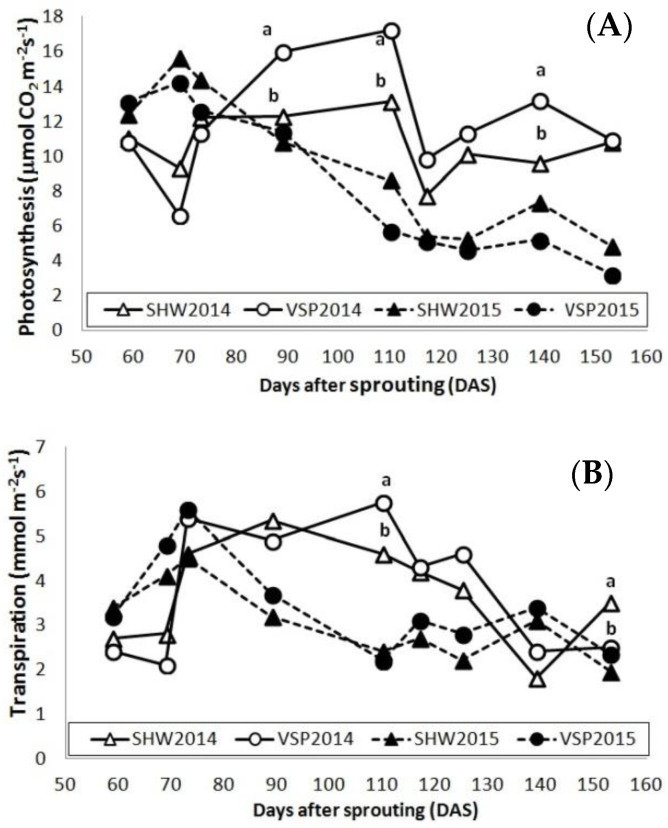
(**A**) Photosynthesis (μmol m^−2^s^−1^) and (**B**) transpiration (mmol m^−2^s^−1^) in 2014 (white circle, vertical shoot positioning, VSP; white triangle, single high wire, SHW) and in 2015 (black circle, VSP; black triangle, SHW). Data (mean, *n* = 45) were subjected to one-way ANOVA. Different letters (a, b) within the same year (solid line, 2014; dashed line, 2015) and days after sprouting (DAS) indicate significant differences between VSP and SHW (LSD test, *p* ≤ 0.05).

Figure 2 shows the seasonal trends of photosynthesis and transpiration rates for VSP and SHW systems in 2014 and 2015. In general, these rates were higher in 2014 than in 2015 because of milder temperatures and higher rainfall amounts (Figure 1). In addition, in both 2014 and 2015, the downward trends of gas exchanges observed in both trellis systems are in line with a progressive and physiological leaf aging [38].

In 2014, significant differences between treatments were found in the mid–final part of the season (90–150 days after sprouting (DAS), July–August), evidencing an increase in photosynthesis (23–25% depending on sampling time) and transpiration (17–28% depending on sampling time) for VSP when compared with SHW. This lower net assimilation rate in SHW around veraison is linked to the shading of mature and fully functional leaves (8–10 nodes), which is caused by the typical falling vegetation of this training system [37,38,39,40]. Moreover, in 2014, lower values of photosynthesis and transpiration were detected from 115 DAS onwards (August) in comparison with the period between 90 and 110 DAS (July) due to lower rainfall amounts in August than in July (Figure 1).

On the contrary, in 2015, no significant differences were found between the two treatments.

The seasonal average of the photosynthetic active radiation intercepted by the canopy was around 16% in VSP and 18% in SHW in both years (data not shown), with no statistical differences between treatments.

### 2.2. Vegetative Growth, Dry Matter Measurements and Yield Components

**Figure 3:**
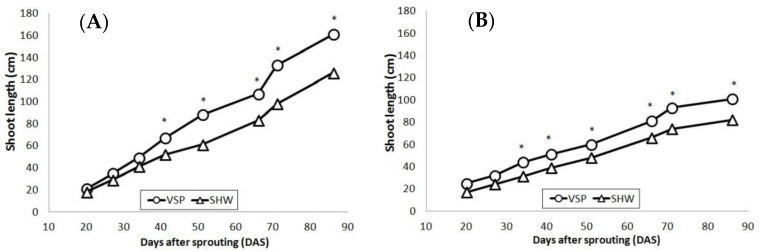
Shoot length (cm) in (**A**) 2014 (circle, vertical shoot positioning, VSP; triangle, single high wire, SHW) and (**B**) 2015 (circle, VSP; triangle, SHW). Data (mean, *n* = 45) were subjected to one-way ANOVA. Asterisks * within the same year and DAS indicate significant differences among VSP and SHW (LSD test, *p* ≤ 0.05).

As shown in Figure 3, a constant shoot growth up to the date of topping (86 DAS) was recorded for the two training systems in both years, with higher values in 2014 for both training systems (VSP reached 160 cm, while SHW 120 cm) than 2015 (100 cm for VSP and 80 cm for SHW). From the statistical analysis of data, a significant difference between the two treatments from the third sampling date up to trimming was highlighted in both years, with higher values for VSP than SHW in both years (+25% in 2014, +20% in 2015). A greater shoot elongation for the traditional vertical shoot positioning trellis was also observed in other works [41]. Higher values for VSP were also found in the lateral leaf area in both years, despite the total leaf area being greater in SHW than VSP in both years (Table 1).

No statistical differences were evidenced for the cluster number and berry weight, whereas the cluster weight was always higher in SHW than VSP. This suggested a higher number of berries/clusters in SHW, which was probably determined by a better fruit set and/or a smaller berry drop after fruit set as a result of the greater cluster shading in the SHW canopy [42,43]. The yield ranged from +9% (2014) to +11% (2015) in SHW compared to VSP, primarily as a function of cluster weight, in accordance with the results of other authors [37]. Moreover, the vine size in this trial was relatively small overall, with pruning weights around 0.4–0.5 kg/vine (data not shown), indicating that vines were moderately vigorous on this site; nevertheless, both training systems were quite balanced in terms of yield and pruning weight, as shown by the Ravaz index (Table 1).

### 2.3. Berry Composition

Concerning the technological maturity (Table 2), statistical differences were noticed only in 2015, probably because the rainy period before the harvest in 2014 flattened the differences between the two trellis systems. In 2015, the lower values of sugar content and pH and the higher total acidity in SHW than VSP suggested a slower ripening of the grapes in the SHW, which was related to the lower photosynthesis during berry ripening (Figure 2A). This result has also been demonstrated by other authors, who observed a general decrease in carbon fixation and a consequent reduction in the content of sugars in the same trellis system linked to the shading of basal leaves and clusters by the falling vegetation [42,44,45,46,47,48]. The higher cluster weight and yield for the SHW (Table 1) could justify a decrease in the accumulation of sugars, as demonstrated in other works in which higher production was associated with lower quality [49].

In a similar manner to the technological maturity, phenolic compounds only showed significant differences between treatments in 2015 (Table 2). In this year, it was possible to observe greater total polyphenol concentrations in SHW, whereas higher total anthocyanins were detected in VSP. Considering that the phenylpropanoid pathway produces polyphenols as precursors and culminates in the synthesis of anthocyanins [50,51], we hypothesized a different timing in the accumulation of secondary compounds in the two trellis systems: the VSP, which had a more accelerated maturation, reached the final stages of the biosynthetic pathway at harvest (i.e., higher total anthocyanins and lower total polyphenols), while the SHW, which had a delayed ripening, was not at the peak of the biosynthetic pathway at the time of harvest, as shown by a higher total content of polyphenols (i.e., precursors) but minor content of total anthocyanins. The extractability of polyphenols was in any case lower in the SHW than in VSP, probably because polyphenols synthesized in the skin were not yet mature (e.g., condensed tannins) and therefore less extractable [52].

Although these results for berry composition were supported by statistical significance only in 2015, the same trends were also observed in 2014. Therefore, summing up the qualitative parameters of the two trellis systems, it is possible to state that SHW showed a better balance between technological and phenolic maturities than VSP, with lower sugars, higher acidity and a content of secondary compounds typical for Sangiovese cv., together with a higher yield.

### 2.4. Grapevine Model Structure

**Figure 4:**
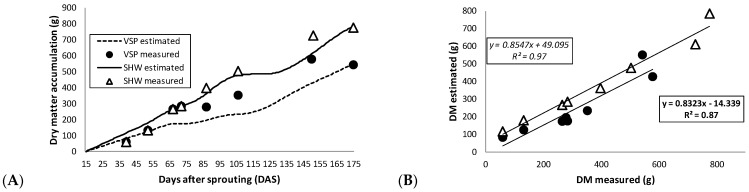
(**A**) STELLA-estimated dry matter accumulation (dashed line, vertical shoot positioning, VSP; solid line, single high wire, SHW) versus measured dry matter accumulation (black circle, VSP; white triangle, SHW). (**B**) Linear regressions (bold, VSP; italic, SHW) between dry matter (DM) measured and estimated with STELLA for VSP (black circle) and SHW (white triangle). Both graphs represent the average measured values for 2014 and 2015 of dry matter accumulation to match the two-year average dry matter estimated by the model. Regression equations and coefficients (R^2^) are shown.

The two-year average total accumulation of dry matter (Figure 4A) was greater for the SHW compared to the VSP (+31%), largely due to a significantly higher yield in SHW (9–11% depending on the year; Table 1). Moreover, a higher correspondence between the STELLA-estimated dry matter and the measured dry matter was recorded for SHW, while the software tended to underestimate dry matter accumulation in VSP in the mid–final season (from 70 DAS on), as previously reported by other authors [28,33]. This could be related to an underestimation of the daily CO_2_ balance, since the model did not take into consideration the photosynthetic contribution of laterals, whose CO_2_ uptake increased in the second part of the season [39]. Consequently, as the lateral leaf area was higher in VSP than SHW (Table 1), the divergence between dry matter values estimated by the STELLA software and those actually measured in the field was greater in VSP compared to SHW.

In spite of this modest underestimation, the coefficients of determination between the dry matter values actually measured in the sampled shoots and the dry matter values estimated by the software in the time of shoot sampling in both trellis systems were significant and positive (R^2^ values of 0.87 and 0.97 in VSP and SHW, respectively) (Figure 4B). In addition, the mean absolute error (MAE) values were low (0.24 for VSP and 0.19 for SHW), the relative root mean square error (RRMSE) was less than 0.7 in VSP and around 0.5 in SHW, and the percentage of bias (PBIAS) surpassed 21% in the case of VSP and 2% in SHW (Table 3).

Hence, as indicated by the goodness-of-fit indicators, STELLA faithfully simulated the accumulation of dry matter in both training systems. Therefore, the model is also calibrated and validated for the accumulation of dry matter for Sangiovese cv. vines trained with an SHW and VSP, confirming and integrating what had been stated by other researchers with other grapevine cultivars [27,28,32,33].

**Figure 5:**
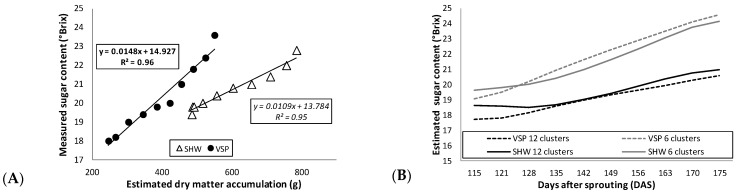
(**A**) Linear regressions (bold, vertical shoot positioning, VSP; italic, single high wire, SHW) between measured sugar content and STELLA-estimated dry matter accumulation for VSP (black circle) and SHW (white triangle). (**B**) Estimated sugar content varying the number of clusters for VSP (black dashed line, 12 clusters; grey dashed line, 6 clusters) and for SHW (black solid line, 12 clusters; grey solid line, 6 clusters). Figure 5A shows the average measured values of sugar content from 2014 and 2015 to match the two-year average dry matter estimated by the model. Regression equations and coefficients (R^2^) are shown.

Figure 5A highlights a more gradual two-year sugar accumulation pattern in SHW compared to VSP, since the sugar content from the first data point to the last increased by 20% in SHW and 31% in VSP. This support the hypothesis of a slower technological maturation in the SHW system, which can be considered as an alternative to VSP in order to obtain grapes with a lower sugar content, higher acidity and a higher content of anthocyanin precursors, as confirmed by other studies [48,53]. Moreover, for the first time, strong linear regressions were found between measured sugar content and estimated dry matter accumulation in both trellis systems (0.96 and 0.95 in VSP and SHW, respectively) (Figure 5A). The linear regressions allowed the model to estimate the berry sugar content from the simulated dry matter. This latter evidence opens up the possibility of varying some crop level parameters in the sub-modules of the software (e.g., number of clusters per vine) to predict the sugar content of the berries as a function of the estimated dry matter accumulation. As an example, Figure 5B shows the predicted trends of the sugar content in both trellis systems by varying the number of clusters in the software with respect to the average of clusters actually measured in our study (i.e., approximately 10 clusters/trellis system; Table 1). With six clusters/vines, an increase in sugar content in both VSP and SHW is observed. Thus, on the contrary, with more clusters/vines (e.g., 12), the sugar content would decrease.

Since traditional analytical methods used in technological measurements of grapes are invasive, costly and time-consuming, non-destructive evaluations have a great practical impact for winemakers in the prediction of berry sugar accumulation (which is directly related to the wine’s alcoholic strength) during the season, allowing them to modulate conventional canopy management practices (e.g., cluster thinning) to obtain the desired sugar content at harvest. Cluster thinning is a widely used practice to optimize the ratio between grape quantity and quality in vineyards [54]. However, the timing and intensity of cluster thinning are not easy to determine because they depend on the vintage and the wine style to be produced. Therefore, the software presented in the current study can support winegrowers in obtaining the optimal balance between grape quality and yield, according to the desired wine style. This could increase the price of wine.

## 3. Materials and Methods

Trials were conducted during the 2014 and 2015 growing seasons in the experimental vineyard of trellis systems at the Montepaldi estate, in the Chianti Classico area (Lat. 43.668° N, Lon. 11.145° E), Tuscany, Italy, located at an elevation of 250 m a.s.l. with south–west exposure. The climate is typically Mediterranean, characterized by rainy winters and dry, warm to hot summers. The soil (2–25 cm of depth) presents a clay loam texture with the following average characteristics: clay, 38.8%; silt, 37.8%; sand, 23.4%; organic matter, 2.0%; pH (H_2_O), 7.8.

The 23-year-old vineyard of the red cv. Sangiovese (*Vitis vinifera* L.), clonal selection R 24, grafted on 420 A rootstock, was planted with a spacing of 1.2 m × 3 m (~2778 vines/ha). Vines were rainfed-cultivated and received, annually, 70 kg/ha of nitrogen in split applications with 35 kg applied between bud-break and bloom, and an additional 35 kg after harvest. In the experimental plot, arranged in a randomized complete block design, consisting of 5 blocks (3 rows each) and one factor (training system), half of the vines were trained with a traditional vertical shoot positioning (VSP) trellis with a single cordon at 80 cm above the ground, and the other half with a single high wire (SHW) trellis, where the single cordon was established at 170 cm above the ground and supported by a single wire (Supplementary Figure S1). Both VSP and SHW trellis were spur pruned with a load of 12 buds per vine distributed over 6 spurs. In both VSP and SHW, shoot trimming to 12 main leaves was performed 86 days after bud-break in both years (25 June and 22 June in 2014 and 2015, respectively). No significant re-growth occurred afterwards.

From the central row of each block, 3 homogeneous vines/block/trellis system (15 vines per trellis system) were randomly tagged and used for leaf gas exchanges, canopy light interception, vegetative growth, biomass, yield and grape composition assessments.

### 3.1. Vineyard Microclimate, Leaf Gas Exchanges and Canopy Light Interception

Total rainfall (mm) and mean air temperature (°C) values were collected daily from April to September (from sprouting to harvest) by an automatic meteorological station (Ecotech, Germany) located 50 m far from the vineyard. The GDD or Amerine and Winkler index [55] was also calculated using a 10 °C base temperature. Briefly, the GDD expresses the sum of all daily temperatures for the active growth in an area during the vine growing season between 1 April and 31 October. A portable infrared gas analyzer (model CIRAS-3, PP-Systems, Amesbury, MA, USA) was used to measure the net photosynthesis (*P_n_*, μmol m^−2^s^−1^) and transpiration (*E*, mmol m^−2^s^−1^) of 3 fully expanded leaves from each tagged vine (9 leaves/block per trellis system, a total of 45 leaves per trellis system). Readings were performed approximately every 10 days, from 60 to 150 DAS, between 08:30 and 10:30 a.m., setting the leaf chamber flow at ambient temperature, at ambient CO_2_ concentration (400 μmol mol^−1^) and at a saturating photosynthetic photon flux of 1300 μmol m^−2^s^−1^. On the same days, at different times during the day (8 a.m., 10 a.m., noon, 2 p.m. and 4 p.m.), the photosynthetic active radiation (PAR, µmol m^−2^s^−1^) intercepted by the canopy was measured with a ceptometer (model LP-80, Decagon Devices, Pullman, WA, USA), from the tagged vines of each block, both for VSP and SHW. Readings were collected horizontally at 10 cm above the ground and every 10 cm along the row length, covering an area of 3.6 m^2^.

### 3.2. Vegetative Growth, Dry Matter Measurements and Yield Components

Three shoots per tagged vine (9 shoots/block per trellis system, a total of 45 shoots per trellis system) were labeled and used to measure shoot elongation (cm) approximately every week, starting from 20 DAS to the shoot trimming (86 DAS). At the same times, three shoots/block/trellis system (a total of 15 shoots per trellis system) were collected randomly from non-tagged vines and transferred to the laboratory, where the leaf area (m^2^) of both main and lateral leaves was determined using the plant image analysis software Tomato Analyzer 3.0 (van der Knaap Lab., Athens, GA, USA). Moreover, the dry matter (g) of the collected shoots was measured after drying at 70 °C in an oven up to constant weight.

Approximately every week, the number of leaves, laterals, clusters and the leaf main vein length (cm) of the leaves of all tagged vines/block/trellis system were determined. The main vein length measured was used to estimate the total vine leaf area (m^2^), according to the method of Carbonneau and Mabrouck [56].

The clusters from tagged vines were individually harvested by hand (11 September and 22 September in 2014 and 2015, respectively), as the must sugar content reached about 23° Brix. Yield per vine (kg) was quantified using a portable electronic scale (Bonso Advanced Technology Ltd., Hong Kong) and the cluster number per vine was counted. Cluster weight (g) was calculated by dividing yield by cluster number on a per vine basis. Moreover, in early January, pruning weights were collected. The Ravaz index [57] was calculated by dividing yield by pruning weight on a per vine basis. Total shoots, base shoots, primary shoots and secondary shoots were counted prior to pruning.

### 3.3. Berry Composition

From veraison to harvest in each season, approximately every week (10 data point per year and trellis system), a 50-berry sample was collected, mixing berries from the three tagged vines of each block of both VSP and SHW vines (5 samples of 50 berries in total per trellis system) to perform technological analyses and determine ripening curves, and thus the optimal maturity level to harvest. Each sample was weighed with a digital scale (PCE Italia s.r.l, Capannori, Italy) and immediately juiced. Sugar content (°Brix) was measured using a refractometer (Atago, Bellevue, WA, USA), must pH was measured using a portable pH meter (Hanna instrument, USA), and titratable acidity (g L^−1^ tartaric acid) was determined on a 10 mL sample by a manual glass burette using 0.1 M NaOH to an endpoint of pH 7.0. Moreover, only at harvest, a duplicate 50 berry sample was picked, mixing berries from the three tagged vines of each block of both VSP and SHW vines (5 samples of 50 berries in total per training system), stored frozen at −20 °C and then processed for phenolic maturity parameters, total and extractable polyphenols and anthocyanins (mg L^−1^), following the method proposed by Glories [58].

### 3.4. Grapevine Model Structure

The STELLA software 7.0.3 (Isee Systems, Lebanon, NH, USA) is a user-friendly simulation programming language that has been conceived for ecological modeling. This software was proposed at the beginning of 1990 to foresee crop growth and development in apple trees [29,30] and was later adapted to grapevine [27,28,31,32]. The major processes simulated are the grapevine’s daily CO_2_ balance and seasonal dry matter accumulation (g per vine), obtained by subtracting daily total respiration from daily total photosynthesis and then by multiplying the resulting daily CO_2_ balance by a carbon/dry matter conversion factor [59].

Photosynthesis and organ respiration are simulated at a daily time-step by two different sub-modules (Supplementary Figure S2) that require meteorological and grapevine description inputs, as described in detail by Poni et al. [28]. Briefly, in the photosynthesis sub-module, maximum and minimum temperatures (°C), total radiation (MJ m^−2^day^−1^) and day lengths are meteorological inputs, while maximum leaf photosynthesis (P_n_ max) (g of CO_2_ m^−2^s^−1^), canopy light interception (%), ground allotted per vine (m^2^), light coefficient extinction (K) and quantum yield (µg of CO_2_ J^−1^) are grapevine description inputs. On these bases, the model calculates the effect of temperature on the rate of daily photosynthesis and the daily total photosynthesis per vine (g of CO_2_ per vine per day), following the Charles–Edwards approach [60]. In the respiration sub-module, maximum and minimum temperatures and day length are meteorological inputs, whereas the grapevine description inputs are the shoot surface (m^2^ per vine), the cluster fresh weight (g), the number of clusters per vine and the increment of leaf area (m^2^). This sub-module is based on Arrhenius’s equation, which computes the daily total respiration per vine (g of CO_2_ per vine per day) as the response of the respiration rate to temperature of each organ (shoot, cluster and leaf) [28]. All the above-mentioned inputs were collected in the field during the two growing seasons, as described in Section 3.1, Section 3.2 and Section 3.3, and are listed in Table 4.

### 3.5. Simulations Performed

To assess the behavior of the grapevine model in different situations and thus strengthen its reliability, it was decided to run the model on two trellis systems (VSP and SHW) and in two growing seasons (2014 and 2015). For this purpose, one simulation/trellis system per season was implemented, for a total of four simulations. Another two simulations/season/trellis system were conducted, varying the number of clusters/vines compared to the original dataset, as shown in Table 4. For 1 simulation/season/trellis system, the model was run with 6 clusters per plant, while for the other system, the model was run with 12 clusters per plant.

### 3.6. Statistical Analysis

The values measured on tagged vines were subjected to an analysis of variance each year using the general linear model procedure of the SPSS statistical package (IBM, Armonk, NY, USA), separating the mean values by Fisher’s least significant difference (LSD, *p* ≤ 0.05). Linear regression analysis was performed to assess possible relationships between grape quality parameters and estimated dry matter using Sigmaplot (Systat, Palo Alto, CA, USA).

A set of goodness-of-fit indicators was used to compare the measured data to the outputs of the model: the coefficient of determination (R^2^), the mean absolute error (MAE), the relative root mean square error (RRMSE) and the percentage of bias (PBIAS). All these indicators were calculated following the procedure of Mirás-Avalos et al. [33].

## 4. Conclusions

The present research tested the possibility of correlating the seasonal dry matter accumulation predicted by the STELLA software with berry sugar content in two training systems. Higher gas exchanges and shoot elongation were evidenced in VSP, but a greater dry matter storage in SHW was found. At harvest, a lower sugar content, higher acidity and higher total polyphenols content, together with a higher yield, were detected in SHW compared with VSP, confirming that the single high wire is a highly productive system alternative to traditional vertical shoot positioning for warm to hot climates.

The STELLA software, as indicated by the goodness-of-fit indicators, was useful to simulate vine dry mass production. Therefore, the model has been calibrated and validated for Sangiovese cv. vines trained both as VSP and SHW. In addition, the correlations between berry sugar content and dry matter accumulation allowed sugar content to be estimated over the growing season for both trellis systems. As regards this implementation, the model has yet to be validated, and a multi-year study is already in progress. The trends of the sugar content when varying the number of clusters in the software were predicted for both trellis systems with the sole purpose of showing the potentiality of the model. However, the first-step evidence presented in this study may be usable for other scientists interested in this topic. The software, once validated, could become a non-destructive and useful tool for winemakers to forecast and modulate, during the season, the sugar content potentially synthesized at harvest and to support decision making in conventional cultivation practices (e.g., cluster thinning). Further experiments will be carried out in order to validate the model in other varieties, training systems and environments and to investigate the possibility of simulating other qualitative parameters; for example, the accumulation of anthocyanins and polyphenols.

## Figures and Tables

**Table 1:** Vegetative growth and yield parameters of Sangiovese grapevines trained to vertical shoot positioning (VSP) and to single high wire (SHW) in 2014 and in 2015 in Tuscany (Italy).

Treatment	Lateral Leaf Area (m^2^)	Total Leaf Area (m^2^)	Cluster Number	Berry Wt (g)	Cluster Wt (g)	Vine Yield (kg)	Ravaz Index
2014							
SHW	1.14	4.24	9.2	2.85	315	2.89	5.38
VSP	1.86	3.87	10.2	2.91	268	2.63	6.47
Significance	*	*	ns	ns	*	*	*
2015							
SHW	1.21	3.78	10.4	2.12	287	2.67	5.86
VSP	1.45	3.25	10.6	2.28	224	2.37	5.95
Significance	*	ns	ns	ns	*	*	ns

Data were subjected to a one-way ANOVA. Asterisks * indicate significant differences between VSP and SHW over the same year (LSD test, *p* ≤ 0.05), and “ns” indicates no significant differences. Other abbreviations are as follows: berry wt—berry weight; cluster wt—cluster weight.

**Table 2:** Parameters of technological and phenolic maturity of Sangiovese grapevines trained with vertical shoot positioning (VSP) and with a single high wire (SHW) in 2014 and in 2015 in Tuscany (Italy). Data (*n* = 5) were subjected to a one-way ANOVA. Asterisks indicate significant differences between VSP and SHW over the same year (LSD test, *p* ≤ 0.05), and “ns” indicates no significant differences. Other abbreviations are as follows: TA—total acidity, total polyph—total polyphenols, extr polyph—extractable polyphenols, total anth—total anthocyanins, extr anth—extractable anthocyanins.

Treatment	Sugar Content (°Brix)	pH	TA (g L^−1^)	Total Polyph (mg L^−1^)	Extr Polyph (mg L^−1^)	Total Anth (mg L^−1^)	Extr Anth (mg L^−1^)
2014							
SHW	22.5	3.18	5.75	1964	1345	966	449
VSP	22.7	3.21	5.67	1899	1386	987	451
Significance	ns	ns	ns	ns	ns	ns	ns
2015							
SHW	23.2	3.28	4.89	2320	1564	1047	685
VSP	24.5	3.37	4.21	2187	1823	1182	679
Significance	*	*	*	*	*	*	ns

**Table 3:** Indicators of model performance: the coefficient of determination R^2^; MAE, mean absolute error; RRMSE, relative root mean square error and PBIAS, percentage of bias for Sangiovese grapevines trained with vertical shoot positioning (VSP) and with a single high wire (SHW).

Indicator	VSP	SHW
R^2^	0.87	0.97
MAE	0.23	0.19
RRMSE	0.67	0.53
PBIAS	21%	2%

**Table 4:** Model inputs, units and values for vertical shoot positioning (VSP) and single high wire (SHW) in 2014 and in 2015.

Type of Input	Input	Unit	Value 2014		Value 2015	
			VSP	SHW	VSP	SHW
Meteorological	Maximum temperature	°C	12.4–37.7		13.6–34.7	
	Minimum temperature	°C	6.3–21.5		2–18.3	
	Day length	s	41,940–56,040		44,220–56,040	
	Total radiation	MJ m^−2^day	4.76–44.56		4.24–28.97	
Grapevine description in the Photosynthesis sub-module	P_n_ max	g m^−2^s^−1^	0–0.001	0–0.0009	0–0.0008	0–0.0008
	Canopy light interception	%	1–16	1–18	1–16	1–18
	Light extinction coeff. (K)	dimensionless	0.5–0.6	0.5–0.6	0.5–0.6	0.5–0.6
	Ground allotted per vine	m^2^	3.6		3.6	
	Quantum yield	µgCO_2_J^−1^	0.67–3.90		0.67–3.90	
Grapevine description in the Respiration sub-module	Shoot surface per vine	m^2^	0–0.16	0–0.25	0–0.15	0–0.21
	Total shoots per vine	*n*°	12		12	
	Cluster fresh weight	g	0–268	0–315	0–224	0–287
	Clusters per vine	*n*°	10.2	9.2	10.6	10.4
	Leaf area (LA)	m^2^	0–3.9	0–4.2	0–3.2	0–3.8

## Data Availability

Data is contained within the article.

## Supplementary Materials

The following are available online at https://www.mdpi.com/article/10.3390/plants10081675/s1, Figure S1: Experimental site pictures, Figure S2: simplified model structure of STELLA software.
