# Supplementary material for: Modeling Carbon Balance and Sugar Content of Vitis vinifera under Two Different Trellis Systems
Source: Plants (Basel). 2021 Aug 15;10(8):1675. doi: 10.3390/plants10081675 (PMC8402180; doi:10.3390/plants10081675)
Supplement: Supplementary file 1 [file plants-10-01675-s001.zip › plants-1326266-supplementary.pdf]

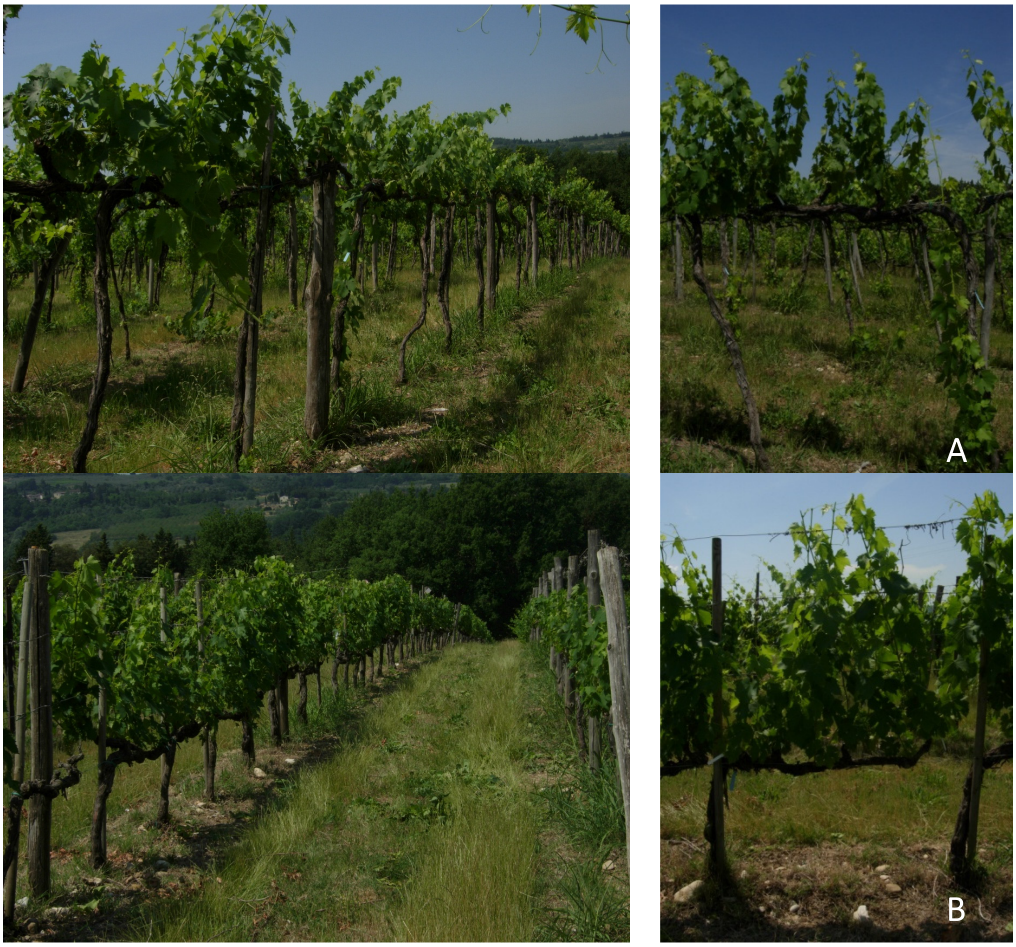

Supplementary Figure S1. Experimental site pictures. (**A**) SHW trellis, (**B**) VSP trellis.

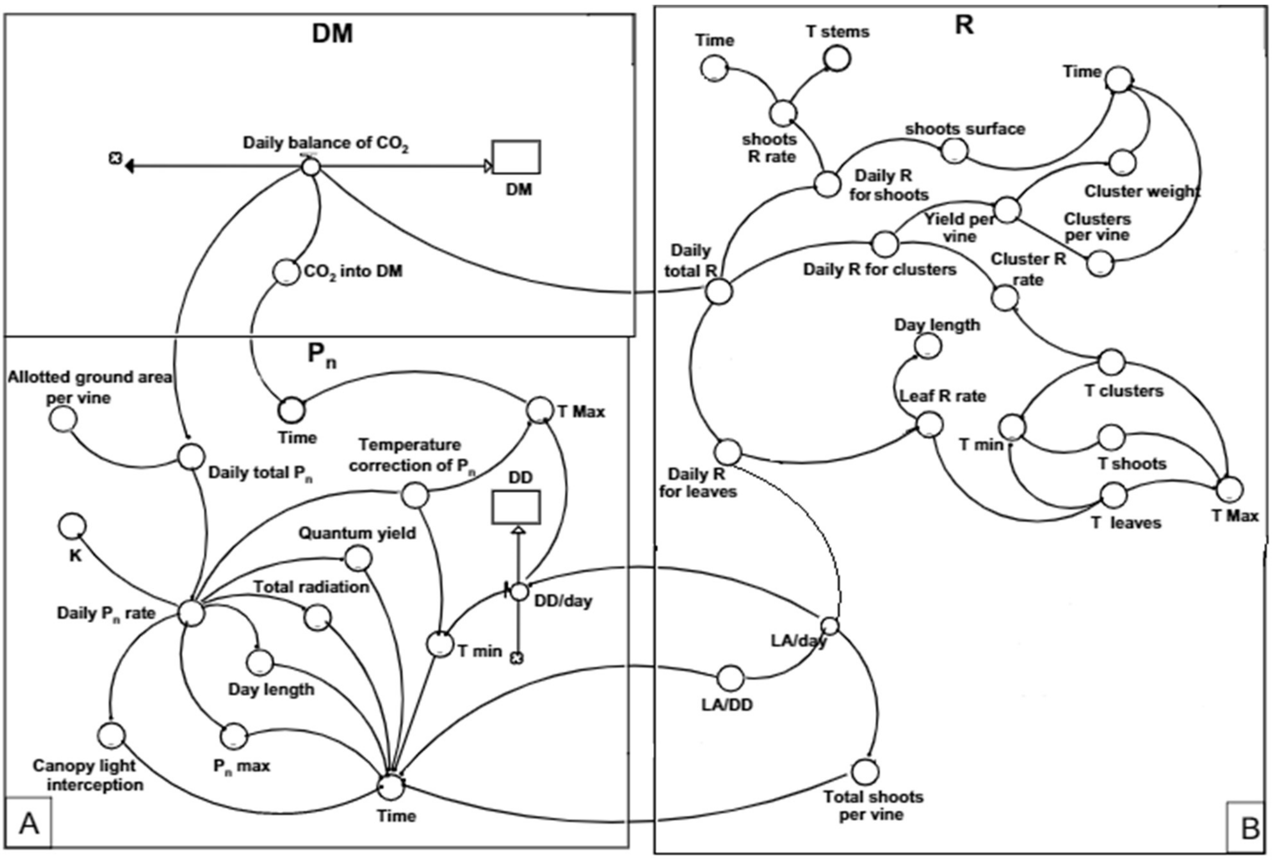

Supplementary Figure S2. Simplified model structure of STELLA software for prediction of the daily balance of CO_2 and dry matter (DM) in grapevine canopies (**A**) Photosynthesis sub-module (P_n). (**B**) respiration sub-module (R). Abbreviations: maximum leaf photosynthesis (P_n max); canopy light extinction coefficient (K); degree days (DD); minimum and maximum daily temperature (T min and T max); temperature (T); leaf area formed per DD (LA/DD). Figure modified by Poni et al. [28]. For other abbreviations, input description and details about building blocks refer also to STELLA software manual 7.0.3 (Isee Systems, N.H.).
